# Prévalence et caractéristiques de l'automédication chez les étudiants de 18 à 35 ans résidant au Campus de la Kasapa de l'Université de Lubumbashi

**DOI:** 10.11604/pamj.2015.21.107.5651

**Published:** 2015-06-09

**Authors:** Valentin Bashige Chiribagula, Henry Manya Mboni, Salvius Bakari Amuri, Grégoire Sangwa kamulete, Joh Kahumba Byanga, Pierre Duez, Jean Baptiste Lumbu Simbi

**Affiliations:** 1 Laboratoire de Pharmacognosie, Faculté des Sciences Pharmaceutiques Université de Lubumbashi, 27av Kato commune Kampemba, Lubumbashi/RD Congo; 2 Laboratoire de Chimie Thérapeutique et Pharmacognosie, Faculté de Médecine et de Pharmacie, Université de Mons (UMONS), bâtiment Mendeleiev, Av Maistriau, 7000 Mons, Belgique; 3 Laboratoire de chimie Organique, Faculté des Sciences Université de Lubumbashi, 11 Av Likasi Commune de Lubumbashi /RD Congo

**Keywords:** Automédication, étudiants, Université de Lubumbashi, self-medication, students, University of Lubumbashi

## Abstract

**Introduction:**

L'automédication est devenue un phénomène émergeant et menaçant de plus en plus la santé publique. La présente étude objective de déterminer la prévalence et les caractéristiques dans le campus Universitaire Kasapa de l'Université de Lubumbashi.

**Méthodes:**

L'interview indirecte a servi à la collecte des données qui ont été traitées par le logiciel Graphpad version 5.

**Résultats:**

De 515 étudiants consultés, l'automédication présente une prévalence de 99%, une partie des sujets l'ayant débutée à l'adolescence (35%). Des répondants, 78,8% reconnaissent que l'automédication peut conduire à un échec thérapeutique et que des erreurs de dose, un traitement inadapté, des effets secondaires et des erreurs diagnostiques sont plausibles. Cette pratique est acceptée pour autant qu'elle permette de prendre en charge des maladies ou symptômes présumés bénins et connus avec pour avantages, discrétion et économie de temps et d'argent. La malaria (82,4%), la fièvre (65,5%), les maux de tête (65,5%) en constituent les trois premières causes. L'amoxicilline (98,2%), le paracétamol (97,5%), l'acide ascorbique (91,6%) et la quinine (79,4%) sont les quatre premiers médicaments les plus consommés. L'association la plus utilisée est paracétamol’ vitamine(s) (88,8%) et la plus aberrante amoxycilline -Erytromycine (25,5%). Le comprimé (37%) constitue la forme la plus utilisée. La plupart des sujets (84,9%), recourent aux plantes médicinales.

**Conclusion:**

Dans ce milieu, il existe une forte prévalence de l'automédication largement dans un but antipalustre avec quelques abus.

## Introduction

Selon l'OMS, l'automédication consiste dans le fait qu'un individu recoure à un médicament, de sa propre initiative ou de celle d´un proche, dans le but de soigner une affection ou un symptôme qu'il a lui-même identifié, sans avoir recours à un professionnel de santé [1]. L´automédication peut concerner aussi bien la médecine moderne que la médecine traditionnelle [2, 3]. Considérée comme un phénomène menaçant de plus en plus la santé de la population des travaux qui s'y sont intéressés ont insisté sur les dérives qui peuvent en découler [4–16] en soulignant les principaux risques, plausibles ou avérés, notamment les résistances microbiennes acquises envers les médicaments, les accidents médicamenteux, les interactions médicamenteuses non bénéfiques, la pharmacodépendance et la toxicomanie [5, 16–20]. En Afrique, plusieurs travaux ont présenté les prévalences [12–14] ainsi que les caractéristiques de l´automédication, constituées essentiellement de ses motivations (coût élevé de la prise en charge des malades dans les formations sanitaires, faible pouvoir d´achat, insuffisance en infrastructures et personnels sanitaires, banalisation de certaines maladies, complicité de certains vendeurs en pharmacie ne respectant pas les règles de délivrance des médicaments et absence d´information et de sensibilisation sur les risques liés aux mauvais usages des médicaments [2, 3, 15]); et de ses méfaits (non-maîtrise des indications, des contre-indications, des posologies, des rythmes d´administration et de la durée du traitement). En RDCongo, la prévalence de l'automédication a été estimée à 49% en 2001, sur l'ensemble de la population [21], et à 57% à Goma en 2013 [22]. A Lubumbashi les données relatives à la prévalence et aux caractéristiques de l'automédication chez les étudiants sont inconnues. La présente étude, réalisée de février à avril 2014 auprès des étudiants de 18 à 35 ans résidant au Campus de la Kasapa de l'Université de Lubumbashi en RD Congo, vise à déterminer la prévalence de l'automédication et ses caractéristiques dans ce milieu.

## Méthodes

**Figure 1:**
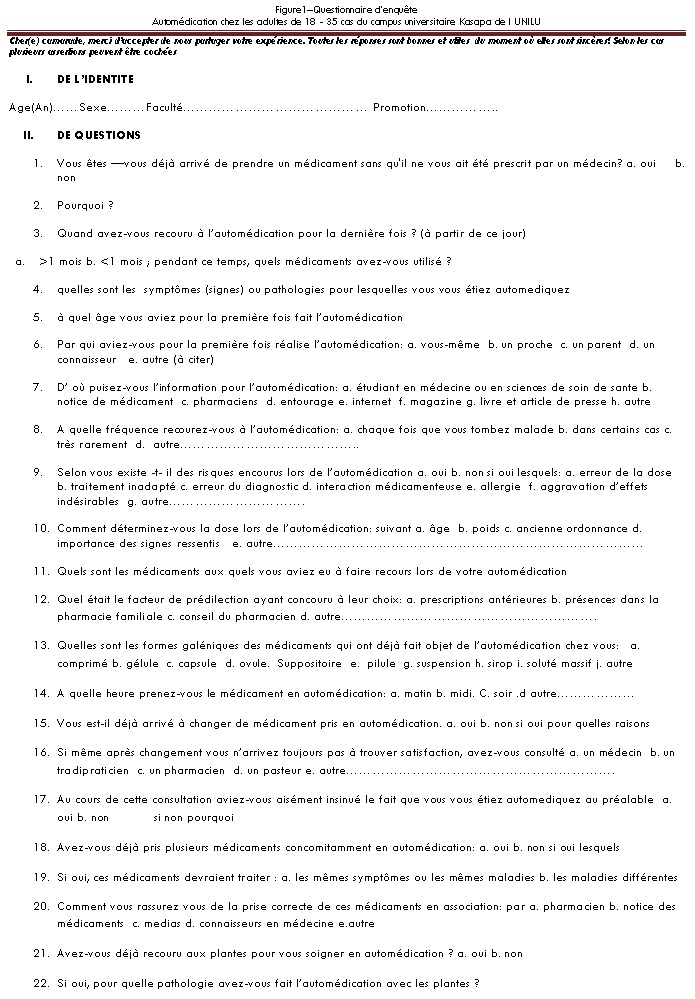
Questionnaire d'enquête

Cette étude descriptive transversale a été menée par une interview indirecte grâce à un questionnaire auprès de 600 étudiants de l’âge variant entre 18 à 35 anset habitant le campus Kasapa de l'Université de Lubumbashi, province du Katanga en République Démocratique du Congo. Ce campus se situe dans la commune de Lubumbashi, dans le quartier Gambela I et loge un total de 4521 étudiants. Nous avons reçu des éléments de réponse de 515 étudiants. Le questionnaire comportait 22 questions, dont certaines ouvertes et d'autres à choix multiples (Figure 1). Le contenu du questionnaire avait fait objet d'un pré-test auprès de 20 sujets de la population cible de manière à nous assurer de la compréhension des questions. Le but de l’étude et la définition de l'automédication ont été précisés aux sujets dans le questionnaire. Les données statistiques ont été calculées en fonction des 510 sujets concernés par l'automédication et sont exprimées en pourcentage. En vue d’établir une comparaison entre les différents groupes des sujets en rapport avec leurs caractéristiques générales, le test ANOVA a été utilisé grâce au logiciel Graph pad version 5. La méthode probabiliste a été utilisée pour l’échantillonnage. La taille de l’échantillon a été déterminée par la formule de Schwartz: n = z_α_2*p*(1-p) /i^2^ [23] avec z_α_=1,96 (écart-type correspondant au risque d'erreur de 5%); p= prévalence de l'automédication (en considérant par défaut la prévalence nationale rapporté, p= 0,49); i= précision souhaitée, fixée à 4,4%;ainsi n = 495,9 qui a été ramené à 600 pour tenir compte d´éventuels retraits de l´étude.

## Résultats

**Figure 2:**
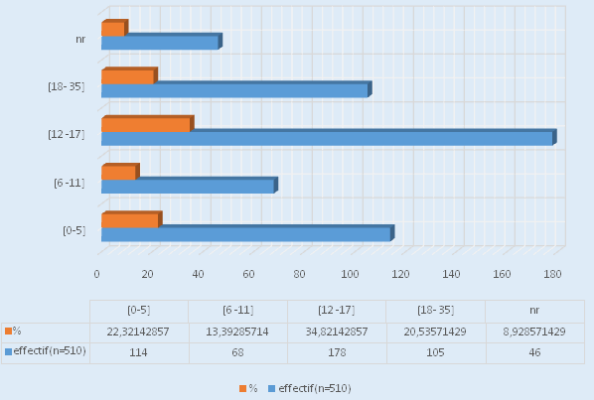
Age de première automédication

Sur 600 questionnaires distribués, 515 ont été retournés (85,8%) qui concernent des étudiants d´âge entre 18 et 35 ans (moyenne: 24,1 ans; médiane: 23 ans), avec un sexe ratio (femme /homme) de 1,1. Dans cette population, l'automédication présente une prévalence de 99%. Les 1% des étudiants qui n´y ont pas recours expliquent cette option par leur non-qualification à décider du choix du traitement approprié et par la peur de commettre des erreurs posologiques (sous- ou surdosage). Parmi les 510 sujets ayant déjà recouru à l'automédication, 230(45,1%) font les sciences de la santé et 280 (54,9%) n'en font pas (Tableau 1). Groupés en 3 classes suivant la conception traditionnelle des étapes de la vie de l'adulte selon Marmor [24], 84% des sujets se situent dans la période "de la fondation" (20 à 30 ans), près de 7% dans les "années fragiles"(10 à 20 ans) et près de 8% dans la période "de famille": 30 à 40 ans. S'agissant de l’âge de la première automédication, les sujets ont été groupés en 4 classes (de 0 à 5 ans, 6 à 11 ans, 12 à 17 ans et 18 à 35 ans) en fonction des étapes du développement psychosocial de la personnalité selon Erickson [25]. Il en découle que 22% des sujets ont commencé leur automédication entre 0 et 5 ans, 35% entre 12 et 17 ans et 21% entre 18 et 35 ans; à cette question, 46 (9%) sujets n´ont pas pu ou voulu répondre (Figure 2).

**Tableau 1:** Caractéristiques générales des sujets

variable		Sciences de la santé (n = 230; 45,1%)		Autres sciences (n = 280; 54,9%)		Total: n = 510(%)	p (IC95%)
		1er cycle (n = 129)	2e cycle (n = 101)	1er cycle (n = 195)	2e cycle (n = 85)		
sexe (ni,%)	Masculin	58 (45)	51 (50,5)	70 (35,9)	63 (74,1)	242 (47,5)	"P > 0,05"
	Féminin	71 (55)	50 (49,5)	125 (64,1)	22 (25,9)	268 (52,5)	"P < 0,05"
âge	[10-20]	13 (10,1)	0 (0)	22 (11,3)	0 (0)	35 (6,9)	"P > 0,05"
	[20-30]	107 (82,9)	91 (90,1)	163 (83,6)	69 (81,2)	430 (84,3)	"P < 0,05"
	[30-40]	9 (6,8)	10 (9,9)	10 (5,2)	16 (18,8)	45 (8,8)	"P > 0,05"

IC: intervalle de confiance fixé à 95%; [a-b]: classe d’âge allant de a à b ans

**Figure 3:**
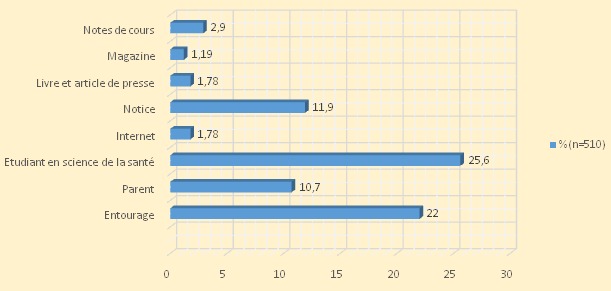
Sources d'information pour l'automédication

**Figure 4:**
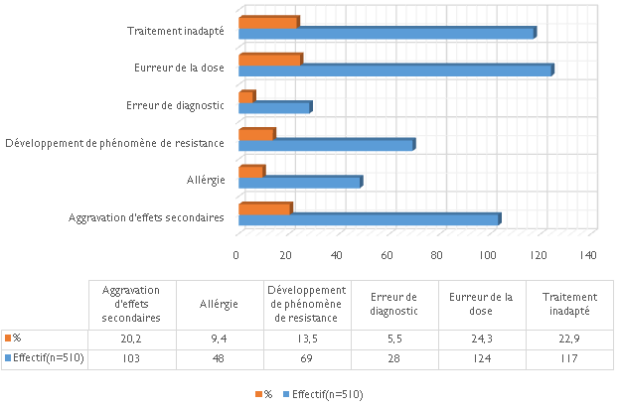
Risques encourus lors de l'automédication

**Figure 5:**
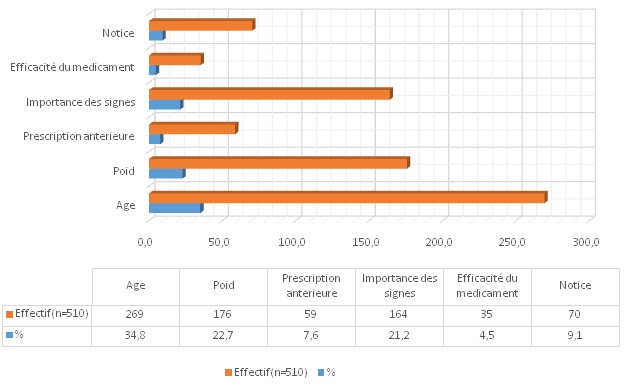
Critères de la détermination de la dose

En ce qui concerne la fréquence d´automédication, 42,2% des sujets consultés y recourent dans certains cas précis seulement, alors que 22,4% y recourent très souvent; 87,5% pensent que les pathologies ou symptômes pour lesquels ils font l'automédication sont sans gravité, 2,1% estiment en connaitre les remèdes les plus efficaces, 42% espèrent ainsi gagner du temps alors que 17,4% se gênent de présenter leur cas devant un médecin ou un pharmacien. Pour ce faire, ils puisent les informations relatives à l'automédication principalement auprès de leurs condisciples étudiants (25, 6%) ou de leur entourage (22%) (Figure 3). La quasi-totalité des sujets (95, 9%) estiment "courir un risque" de par l'automédication; parmi eux, 24,3% estiment que ce risque possible pourrait être lié à une erreur de dose, 23% à un traitement inadapté et 20% à des effets secondaires (Figure 4); 34,8% des sujets déterminent leur dose selon l’âge, 44, 9% selon le poids ou l'importance de signes et 13, 6% selon les instructions de la notice ou l'efficacité prétendue du médicament (Figure 5). Seuls 5,5% estiment que l'erreur de diagnostic est plausible en automédication (Figure 4) alors que 76% des sujets considèrent que le médicament choisi correspond à priori au mal dont ils se plaignent. Cependant, 78,8% affirment s’être présenté occasionnellement à une consultation par suite de l’échec d´une automédication. 17,4% de la population se gène de consulter un personnel de santé. Une fraction de la population (19,2%) note avoir déjà souffert des MST (Tableau 2). Vraisemblablement les sujets se gênent de consulter en cas des MST (maladies sexuellement transmissible).

**Tableau 2:** Pathologies ou symptômes mis en cause lors de l'automédication

Pathologie	Effectif(%) N = 510	Pathologie	Effectif (%) N = 510	Pathologie	Effectif (%) N = 510
Acné	20 (3,9)	Douleurs dentaires	40 (7,8)	Maux de tête	334 (65,5)
Allergie	26 (5,1)	Fièvre	334 (65,5)	MST	98 (19,2)
Amibe	(27,9)	Fièvre typhoïde	316 (62,0)	Mycoses	90 (17,6)
Blessure	30 (5,9)	Gastrite	156 (30,6)	Nausée	36 (7,1)
Constipation	10 (2,0)	Hémorroïde	136 (26,7)	Stimulation sexuelle	50 (9,8)
Diarrhée	40 (7,8)	Infection bactérienne	258 (50,6)	Syndrome grippal	248 (48,6)
Dysménorrhée	86 (16,9)	Insomnie	20 (3,9)	Toux	316 (62,0)
Douleur non dentaires	54 (10,6)	Malaria	420 (82,4)	Vers intestinaux	124 (24,4)
				Vomissement	248 (48,6)

MST: maladie sexuellement transmissible

**Figure 6:**
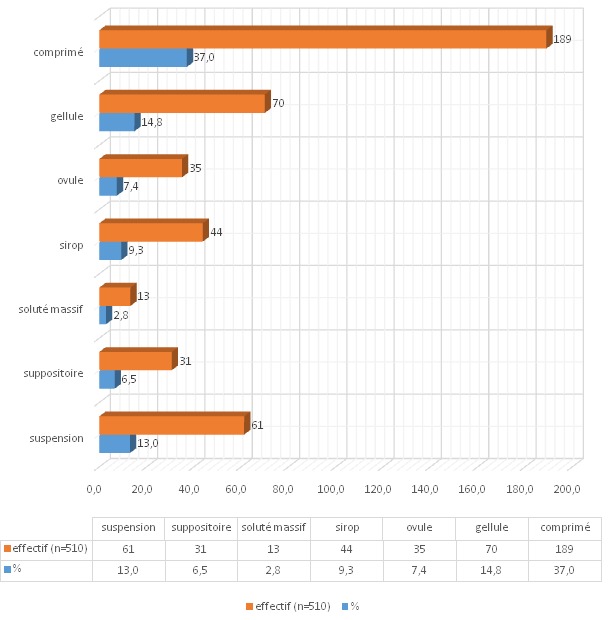
Formes médicamenteuses les plus utilisées

Plusieurs pathologies (20) ont déjà fait l'objet d'automédication par les sujets. La malaria (82,4%), la fièvre (65,5%), les maux de tête (65,5%), la toux (62%) ainsi que les infections bactériennes (50,6%) en constituent les cinq principales causes (Tableau 2). Dans le mois qui a précédé la présente investigation, 48,4% des sujet sont recouru à l'automédication. Parmi eux 40,2% ont recouru à des antibiotiques, tous à un antalgique et 38,6% à un antimalarique. Lors de l´étude, 20,4% étaient en cours d'automédication dont 4,7% pour un anti-infectieux,19,0% pour un antimalarique et 18,4% pour un anti-inflammatoire. Parmi les répondants, 10% utilisent des stimulants sexuels et près de 15% a déjà pratiqué l'automédication pour un syndrome grippal. Plusieurs médicaments sont utilisés en automédication. Amoxycilline (98,2%), paracétamol(97, 5%), vitamine c (91,6%), quinine (79,4%), artéméther (69,4%), aspirine (68,2%), phosphalugel (56,8%) et dihydroartémisinine (56,6%) occupent les huit premières places des médicaments sollicités. Le comprimé (37%) constitue la forme pharmaceutique la plus utilisée (Figure 6). Dans les associations on note: paracétamol-vitamine B(88,8%); amoxycilline-vitamine C (68,6%); amoxycilline-paracétamol (42,7%); ampicilline-multivitamine (42,7%), amoxycilline-érythromycine(25,5%) et ampicilline-gentamicine (0,9%). Un total de 84,9% de la population enquêtée, dont 51,5% sont des femmes, reconnaît avoir déjà recouru aux plantes pour l'auto-prise en charge sanitaire. Parmi les pathologies auto-traitées par phytothérapie figurent la malaria (53,9%), le diabète (43,3%), le cancer (2,4%), l'impuissance sexuelle (1,9%), la pleurésie et la cirrhose du foie (34,9%).

## Discussion

Cette étude réalisée auprès des étudiants résidants au campus Kasapa de l'université de Lubumbashi montre qu'il n'existe pas des différences significative dans le recours à l'automédication entre les étudiants en sciences de la santé et des autres sciences. Cette étude réalisée auprès des étudiants résidants au campus Kasapa de l'université de Lubumbashi montre qu'il n'existe pas des différences significative dans le recours à l'automédication entre les étudiants en sciences de la santé et des autres sciences (p> 0,005). Par contre, il existe une différence significative entre les filles du premier et deuxième cycle d'une part et entre les sujets dont l’âge varie entre [20, 30] ans fréquentant les autres sciences que la science de la santé et ayant participé à l’étude (p < 0,005). A l´instar de cette étude, plusieurs recherches indiquent que l'automédication a tendance à augmenter avec l’âge [26–28] et à être plus fréquente chez les filles que chez les garçons [29–31].

Par ailleurs, les études menées dans les milieux universitaires à Hong Kong [32], au Pakistan [33] au Nigeria [34] et au Brésil [35] avaient révélé des prévalences d'automédication respectivement de 82,3%, 76%, 91,4% et 86,4%. La disparité de l'organisation du système de santé de ces pays associée aux habitudes socio-culturelles pourrait justifier ces taux très variables. Des études menées au Ghanapar Adou [36] et au Sénégal par Ndiaye et al. [15] ont établi que l'automédication se justifie par la "banalisation" des maladies incriminées ainsi que par la prétention de "détention de remèdes appropriés". Les sujets consultés dans la présente étude présentent un avis similaire. La prédominance de l'adolescence comme classe d’âge de première automédication peut se justifier par le fait que, dans cette tranche d’âge, l'individu, à la quête de son identité, d´une conscience, d´une moralité, d´une échelle de valeurs et d´un bien-être, aura tendance à faire de son mieux pour préserver sa santé [25, 36, 37]. Quant à ce qui concerne les taux observés dans la tranche d’âge de la petite enfance, des études antérieures ont révélé, de manière générale, que les taux d'automédication en pédiatrie sont supérieurs à 83% [38–40] et que plus de 40% des parents l'appliquent à leurs enfants dès le bas âge [41]. Une étude menée au Cameroun par Wogaing [42] a établi que 43% des sujets recouraient à l'automédication dans certains cas seulement, des taux identiques à ceux rapportés par la présente étude; une certaine similitude de mœurs entre les 2 populations constitue une explication plausible. Par contre, à Cotonou [12], au Togo [13], à Accra [14], l'automédication occasionnelle représentait respectivement 66,5%, 67,0% et 69,8%. De même pour une étude réalisée en France auprès des parents par Escourou [41], le recours intempestif à l'automédication représentait 50%. La discordance de ces taux à ceux observés par la présente étude peut se justifier par les habitudes socio-culturelles ainsi que le développement du système sanitaire de ces pays. Bien que devenus adultes et vivant loin de leurs parents, près de 11% de sujets les consultent en cas de problèmes sanitaires. Le fait que beaucoup d’étudiants affichent une grande disponibilité à prêter main forte à leurs condisciples, en toute sérénité et sans se préoccuper des risques encourus, est une caractéristique de l'adulte selon Maslow [43].

Une fraction importante des sujets (67,1%) considèrent que le médicament qu'ils prennent correspond au mal dont ils se plaignent. Cependant, 78,8% des sujets affirment avoir été obligés de consulter le médecin par suite de l’échec de l'automédication. Cette discordance peut s´expliquer par le choix soit d´un médicament approprié au mal mais avec une posologie incorrecte, soit d´un médicament inapproprié. Plusieurs travaux avaient déjà fustigé comme abus lors de l'automédication, la non maitrise des indications et des posologies [8, 11, 44]. La haute consommation d´antalgiques, d´anti-infectieux et d´anti malariques laisse envisager que les infections bactériennes et la malaria constituent l'essentiel des causes d'automédication des sujets étudiés. Bon nombre de sujets (95,9%) pratiquent l'automédication en associant plusieurs médicaments. Certaines associations ne se justifient cependant pas. C'est le cas de l'association entre un β-lactame (amoxicilline) et un macrolide (érythromycine). En effet, ce dernier couvre le spectre du premier [44]. La posologie, bien que fruit d´études cliniques poussées [44], ne peut que rarement être respectée de manière optimale, les paramètres pris en compte pour sa détermination n´étant pas exhaustifs. En effet, les posologies mentionnées sur les notices devraient être adaptées suivant les caractéristiques physiques et physio-pathologiques du patient, notamment l´état de nutrition et des fonctions rénales et hépatiques; les paramètres mentionnés par les sujets ne tiennent aucun compte de ces caractéristiques. La malaria, soupçonnée mais le plus souvent non confirmée, apparaît comme la pathologie supposée la plus fréquente (82,4%). Bien que cette parasitémie soit endémique à la région d´étude [45] et la symptomatologie connue de la population, il serait fondamental d´en préciser le diagnostic avant traitement. Cependant, bien souvent, dans l'automédication, la population combat les symptômes plutôt que la cause. Les traitements sont de courte durée, ce qui peut être insuffisant pour un traitement correct et favoriser l´émergence de souches résistantes; la même situation se rencontre au niveau des antibiotiques. Le comprimé est la forme pharmaceutique prédominante (37%). Plusieurs auteurs avaient déjà établi la prépondérance de cette forme dans l'usage routinier des médicaments [44]. Selon l'OMS, 80% de la population mondiale recourt aux plantes pour ses besoins de santé [46]. A Goma, R.D. Congo, une prévalence de 57% a été rapportée dans la population sur le recours à la médecine traditionnelle [22]. Dans la présente étude,84,9% des sujets recourent à l´automédication par les plantes médicinales; la prévalence réelle de l'utilisation des plantes par cette population pourrait néanmoins être bien supérieure puisque cette étude ne relève que des cas de l'automédication.

## Conclusion

Au campus universitaire de la Kasapa, l'automédication est très fréquente avec des abus notables. Elle est prédominée par la médecine non conventionnelle dans le but anti palustre et pratiquée par les étudiants en science autre que celle de la santé en majorité des filles du premier cycle dont l’âge varie entre 20 et 30 ans. Des efforts quant à l'usage rationnel des médicaments méritent d’être conjugués de telle enseigne que s'il parait difficile d’éradiquer l'automédication, qu'elle soit néanmoins contrôlée par les personnels de santé en connivence avec les décideurs politiques. De même, le recours aux plantes demeurant un phénomène émergent, la mise au point d'une interface de concertation entre médecine traditionnelle et biomédecine serait louableAu campus universitaire de la Kasapa, l'automédication est très fréquente avec des abus notables. Elle est prédominée par la médecine non conventionnelle dans le but anti palustre et pratiquée par les étudiants en science autre que celle de la santé en majorité des filles du premier cycle dont l’âge varie entre 20 et 30 ans. Des efforts quant à l'usage rationnel des médicaments méritent d’être conjugués de telle enseigne que s'il parait difficile d’éradiquer l'automédication, qu'elle soit néanmoins contrôlée par les personnels de santé en connivence avec les décideurs politiques. De même, le recours aux plantes demeurant un phénomène émergent, la mise au point d'une interface de concertation entre médecine traditionnelle et biomédecine serait louable.
